# Supplementary material for: Time-related immunomodulation by stressors and corticosterone transdermal application in toads
Source: PLoS One. 2019 Sep 20;14(9):e0222856. doi: 10.1371/journal.pone.0222856 (PMC6754171; doi:10.1371/journal.pone.0222856)
Supplement: S2 Table — Effect of restraint challenge (Exp. 2), captivity duration (Exp. 5) and corticosterone transdermal application (Exp. 6) on plasma corticosterone levels of R. ornata tested through a set of ANOVAs, with plasma corticosterone levels as dependent variable, hour (0, 1, 24h), captivity duration (field, 7, 30, 60 and 90 days), group (control and corticosterone), and time (pre-experiment and post-experiment) and as factors. (DOCX) [file pone.0222856.s002.docx]

**Table S2.** **Corticosterone plasma levels analysis of variance after stressors and corticosterone transdermal application in *R. ornata* toads**. Effect of restraint challenge (Exp. 2), captivity duration (Exp. 5) and corticosterone transdermal application (Exp. 6) on plasma corticosterone levels of *R. ornata* tested through a set of ANOVAs, with plasma corticosterone levels as dependent variable, hour (0, 1, 24h), captivity duration (field, 7, 30, 60 and 90 days), group (control and corticosterone), and time (pre-experiment and post-experiment) and as factors.

| **Experiment** | **Source** | **Type III SS** | **DF** | **MS** | **F** | ***P*** |
| --- | --- | --- | --- | --- | --- | --- |
| **Experiment 2:**  **Restraint**  **0 *vs.* 1 *vs.* 24h**  **(ANOVA)** | Intercept | 29.738 | 1 | 29.738 | 69.811 | **≤ 0.001** |
|  | Error | 2.556 | 6 | 0.426 |  |  |
|  | Hour | 5.602 | 2 | 2.801 | 9.844 | **0.003** |
|  | Error (Hour) | 3.415 | 12 | 0.285 |  |  |
| **Experiment 5:**  **Captivity Duration**  **(univariate ANOVA)** | Intercept | 6.306.965 | 1 | 6.306.965 | 71.278 | **≤ 0.001** |
|  | CD (days) | 5.919.079 | 4 | 1.479.770 | 16.724 | **≤ 0.001** |
|  | Error | 2.566.023 | 29 | 88.484 |  |  |
|  | Total | 19.655.358 | 34 |  |  |  |
|  | Corrected Total | 8.485.102 | 33 |  |  |  |
| **Experiment 6:**  **Corticosterone**  **transdermal application**  **(mixed ANOVA)** | Intercept | 361.406.093 | 1 | 361.406.093 | 63.056 | **≤ 0.001** |
|  | Group | 245.913.361 | 1 | 245.913.361 | 42.905 | **≤ 0.001** |
|  | Error (Group) | 91.704.745 | 16 | 5.731.547 |  |  |
|  | Time | 261.088.671 | 1 | 261.088.671 | 52.050 | **≤ 0.001** |
|  | Time * Group | 220.937.016 | 1 | 220.937.016 | 44.046 | **≤ 0.001** |
|  | Error (Time) | 80.257.486 | 16 | 5.016.093 |  |  |

Abbreviation as follow: **Hour:** 0, 1, 24h; **CD:** Captivity duration; **Group:** Control and corticosterone; **Time:** pre-experiment and post-experiment; **Type III SS:** Type III sum of squares; **DF:** Degrees of freedom; **MS:** Mean square. Variables with *P* significant < 0.05 are highlighted in bold. Experiment details: **Exp. 2:** 0h *vs*. 1h *vs*. 24h restraint; **Exp. 5:** field *vs*. 7 *vs*. 30 *vs*. 60 *vs*. 90 days in captivity; **Exp. 6:** corticosterone transdermal application.
