# Supplementary material for: Time-related immunomodulation by stressors and corticosterone transdermal application in toads
Source: PLoS One. 2019 Sep 20;14(9):e0222856. doi: 10.1371/journal.pone.0222856 (PMC6754171; doi:10.1371/journal.pone.0222856)
Supplement: S3 Table — Effect of corticosterone transdermal application (Exp. 6) on plasma corticosterone levels of R. ornata tested through a set of mixed ANOVAs, with plasma corticosterone levels as dependent variable and group (control, placebo and corticosterone) and time (pre-experiment and post-experiment) as factors. (DOCX) [file pone.0222856.s003.docx]

**Table S3.** **Corticosterone plasma levels analysis of variance after corticosterone transdermal application in *R. ornata* toads**. Effect of corticosterone transdermal application (Exp. 6) on plasma corticosterone levels of *R. ornata* tested through a set of mixed ANOVAs, with plasma corticosterone levels as dependent variable and group (control, placebo and corticosterone) and time (pre-experiment and post-experiment) as factors.

| **Source** | **Type III SS** | **DF** | **MS** | **F** | ***P*** |
| --- | --- | --- | --- | --- | --- |
| Intercept | 207481.734 | 1 | 207481.734 | 35.801 | **≤ 0.001** |
| Group | 248464.924 | 2 | 124232.462 | 21.436 | **≤ 0.001** |
| Error (Group) | 92726.785 | 16 | 5795.424 |  |  |
| Time | 139681.025 | 1 | 139681.025 | 27.155 | **≤ 0.001** |
| Time * Group | 221159.878 | 2 | 110579.939 | 21.497 | **≤ 0.001** |
| Error (Time) | 82302.054 | 16 | 5143.878 |  |  |

Abbreviation as follow: **Group:** Control, placebo and corticosterone; **Time:** pre-experiment and post-experiment; **Type III SS:** Type III sum of squares; **DF:** Degrees of freedom; **MS:** Mean square. Variables with *P* significant < 0.05 are highlighted in bold. Experiment details: **Exp. 6:** corticosterone transdermal application.
