# Supplementary material for: Time-related immunomodulation by stressors and corticosterone transdermal application in toads
Source: PLoS One. 2019 Sep 20;14(9):e0222856. doi: 10.1371/journal.pone.0222856 (PMC6754171; doi:10.1371/journal.pone.0222856)
Supplement: S4 Table — Bonferroni comparisons of mixed ANOVAs for corticosterone transdermal application (Exp. 6) on plasma corticosterone levels of R. ornata, with plasma corticosterone levels as dependent variable and group (control, placebo and corticosterone) and time (pre-experiment and post-experiment) as factors. (DOCX) [file pone.0222856.s004.docx]

**Table S4.** **Corticosterone plasma levels analysis of variance after stressors and corticosterone transdermal application in *R. ornata* toads with Bonferroni comparisons**. Bonferroni comparisons of mixed ANOVAs for corticosterone transdermal application (Exp. 6) on plasma corticosterone levels of *R. ornata*, with plasma corticosterone levels as dependent variable and group (control, placebo and corticosterone) and time (pre-experiment and post-experiment) as factors.

| **Within Comparisons** | | **I** | **J** | **MD (I-J)** | **SE** | ***P*** |
| --- | --- | --- | --- | --- | --- | --- |
| **Time** | Pre-Experiment | Control | Placebo | -4.389 | 5.854 | 1.000 |
|  |  |  | Experimental | -11.120 | 5.221 | 0.147 |
|  |  | Placebo | Control | 4.389 | 5.854 | 1.000 |
|  |  |  | Experimental | -6.732 | 5.512 | 0.719 |
|  |  | Experimental | Control | 11.120 | 5.221 | 0.147 |
|  |  |  | Placebo | 6.732 | 5.512 | 0.719 |
|  | Post Experiment | Control | Placebo | -17.757 | 63.062 | 1.000 |
|  |  |  | Experimental | -326.059 | 56.244 | **< 0.001** |
|  |  | Placebo | Control | 17.757 | 63.062 | 1.000 |
|  |  |  | Experimental | -308.302 | 59.371 | **< 0.001** |
|  |  | Experimental | Control | 326.059 | 56.244 | **< 0.001** |
|  |  |  | Placebo | 308.302 | 59.371 | **< 0.001** |
| **Group** | Control | Pre-Experiment | Post Experiment | -14.102 | 41.408 | 0.738 |
|  | Placebo | Pre-Experiment | Post Experiment | -27.470 | 45.360 | 0.553 |
|  | Experimental | Pre-Experiment | Post Experiment | -329.040 | 35.860 | **< 0.001** |

Abbreviation as follow: **Time:** pre-experiment and post-experiment; **Group:** Control and corticosterone; **MD:** Mean difference between I and J. Variables with *P* significant < 0.05 are highlighted in bold. Experiment details: **Exp. 6:** corticosterone transdermal application.
