# Supplementary material for: Time-related immunomodulation by stressors and corticosterone transdermal application in toads
Source: PLoS One. 2019 Sep 20;14(9):e0222856. doi: 10.1371/journal.pone.0222856 (PMC6754171; doi:10.1371/journal.pone.0222856)
Supplement: S5 Table — Effect of restraint challenge (Exp. 1) and captivity (Exp. 3 and 4) on neutrophil: lymphocyte ratio tested through repeated measures student-t tests on R. ornata. (DOCX) [file pone.0222856.s005.docx]

**Table S5. Neutrophil: lymphocyte ratio student-t test after stressors in *Rhinella ornata* toads.** Effect of restraint challenge (Exp. 1) and captivity (Exp. 3 and 4) on neutrophil: lymphocyte ratio tested through repeated measures student-t tests on *R. ornata*.

| **Experiment** | **T** | **DF** | ***P*** |
| --- | --- | --- | --- |
| Experiment 1: Restraint (Field *vs.* 24h) | -2.76 | 8 | **0.025** |
| Experiment 3: Captivity (Field *vs.* 7 days) | 1.051 | 5 | 0.341 |
| Experiment 4: Captivity (Field *vs.* 30 days) | 0.650 | 6 | 0.540 |

Abbreviation as follow: **DF:** Degrees of freedom. Variables with *P* significant < 0.05 are highlighted in bold. Experiment details: **Exp. 1:** baseline vs. 24h restraint; **Exp. 3:** field *vs*. 7 days in captivity; **Exp. 4:** field *vs*. 30 days in captivity.
