# Supplementary material for: Time-related immunomodulation by stressors and corticosterone transdermal application in toads
Source: PLoS One. 2019 Sep 20;14(9):e0222856. doi: 10.1371/journal.pone.0222856 (PMC6754171; doi:10.1371/journal.pone.0222856)
Supplement: S6 Table — Effect of corticosterone transdermal application (Exp. 6) on neutrophil: lymphocyte ratio of R. ornata tested through a set of mixed ANOVAs, with neutrophil lymphocyte ratio as dependent variable and group (control and corticosterone) and time (pre-experiment and post-experiment) as factors. (DOCX) [file pone.0222856.s006.docx]

**Table S6.** **Neutrophil: lymphocyte ratio analysis of variance after corticosterone transdermal application in *R. ornata* toads**. Effect of corticosterone transdermal application (Exp. 6) on neutrophil: lymphocyte ratio of *R. ornata* tested through a set of mixed ANOVAs, with neutrophil lymphocyte ratio as dependent variable and group (control and corticosterone) and time (pre-experiment and post-experiment) as factors.

| **Source** | **Type III SS** | **DF** | **MS** | **F** | ***P*** |
| --- | --- | --- | --- | --- | --- |
| Intercept | 3.042 | 1 | 3.042 | 49.183 | **≤ 0.001** |
| Group | 0.139 | 1 | 0.139 | 2.251 | 0.151 |
| Error (Group) | 1.113 | 18 | 0.062 |  |  |
| Time | 0.203 | 1 | 0.203 | 5.441 | **0.031** |
| Time * Group | 0.002 | 1 | 0.002 | 0.043 | 0.838 |
| Error (Time) | 0.672 | 18 | 0.037 |  |  |

Abbreviation as follow: **Group:** Control and corticosterone; **Time:** pre-experiment and post-experiment; **Type III SS:** Type III sum of squares; **DF:** Degrees of freedom; **MS:** Mean square. Variables with *P* significant < 0.05 are highlighted in bold. Experiment details: **Exp. 6:** corticosterone transdermal application.
