# Supplementary material for: Time-related immunomodulation by stressors and corticosterone transdermal application in toads
Source: PLoS One. 2019 Sep 20;14(9):e0222856. doi: 10.1371/journal.pone.0222856 (PMC6754171; doi:10.1371/journal.pone.0222856)
Supplement: S7 Table — Effect of restraint challenge (Exp. 1) and captivity (Exp. 3 and 4) on bacterial killing ability tested through Wilcoxon Signed Ranks test on R. ornata. (DOCX) [file pone.0222856.s007.docx]

**Table S7.** **Plasma bacterial killing ability student-t test after stressors in *Rhinella ornata* toads.** Effect of restraint challenge (Exp. 1) and captivity (Exp. 3 and 4) on bacterial killing ability tested through Wilcoxon Signed Ranks test on *R. ornata*.

| **Experiment** | **Z** | **N** | ***Asymp. Sig. (2-tailed)*** |
| --- | --- | --- | --- |
| Experiment 1: Restraint (Field *vs.* 24h) | -1.362 | 10 | 0.173 |
| Experiment 3: Captivity (Field *vs.* 7 days) | -0.674 | 7 | 0.500 |
| Experiment 4: Captivity (Field *vs.* 30 days) | -0.981 | 8 | 0.326 |

Abbreviation as follow: **DF:** Degrees of freedom. Variables with *P* significant < 0.05 are highlighted in bold. Experiment details: **Exp. 1:** baseline vs. 24h restraint; **Exp. 3:** field *vs*. 7 days in captivity; **Exp. 4:** field *vs*. 30 days in captivity.
