# Supplementary material for: Time-related immunomodulation by stressors and corticosterone transdermal application in toads
Source: PLoS One. 2019 Sep 20;14(9):e0222856. doi: 10.1371/journal.pone.0222856 (PMC6754171; doi:10.1371/journal.pone.0222856)
Supplement: S8 Table — Effect of restraint challenge (Exp. 2), captivity duration (Exp. 5) and corticosterone transdermal application (Exp. 6) on bacterial killing ability of R. ornata tested through a set of ANOVAs, with bacterial killing ability as dependent variable, hour (0, 1, 24h), captivity duration (field, 7, 30, 60 and 90 days), group (control and corticosterone), and time (pre-experiment and post-experiment) as factors. (DOCX) [file pone.0222856.s008.docx]

**Table S8.** **Plasma bacterial killing ability analysis of variance after stressors and corticosterone transdermal application in *R. ornata* toads**. Effect of restraint challenge (Exp. 2), captivity duration (Exp. 5) and corticosterone transdermal application (Exp. 6) on bacterial killing ability of *R. ornata* tested through a set of ANOVAs, with bacterial killing ability as dependent variable, hour (0, 1, 24h), captivity duration (field, 7, 30, 60 and 90 days), group (control and corticosterone), and time (pre-experiment and post-experiment) as factors.

| **Experiment** | **Source** | **Type III SS** | **DF** | **MS** | **F** | ***P*** |
| --- | --- | --- | --- | --- | --- | --- |
| **Experiment 2:**  **Restraint**  **0 vs 1 vs 24h**  **(ANOVA)** | Intercept | 8.460 | 1 | 8.460 | 32.513 | **0.002** |
|  | Error | 1.301 | 5 | 0.260 |  |  |
|  | Hour | 0.017 | 1 | 0.013 | 0.139 | 0.787 |
|  | Error (Hour) | 0.608 | 7 | 0.093 |  |  |
| **Experiment 5:**  **Captivity Duration**  **(univariate ANOVA)** | Intercept | 121.141.373 | 1 | 121.141.373 | 184.401 | **≤ 0.001** |
|  | CD (days) | 12.739.986 | 4 | 3.184.996 | 4.848 | **0.006** |
|  | Error | 15.109.729 | 23 | 656.945 |  |  |
|  | Total | 175.896.000 | 28 |  |  |  |
|  | Corrected Total | 27.849.714 | 27 |  |  |  |
| **Experiment 6:**  **Corticosterone transdermal application**  **(mixed ANOVA)** | Intercept | 36.377.607 | 1 | 36.377.607 | 15.744 | **≤ 0.001** |
|  | Group | 4.513 | 1 | 4.513 | 0.002 | 0.965 |
|  | Error (Group) | 36.968.248 | 16 | 2.310.516 |  |  |
|  | Time | 333.336 | 1 | 333.336 | 0.305 | 0.588 |
|  | Time * Group | 236.099 | 1 | 236.099 | 0.216 | 0.648 |
|  | Error (Time) | 17.473.263 | 16 | 1.092.079 |  |  |

Abbreviation as follow: **Hour:** 0, 1, 24h; **CD:** Captivity duration; **Group:** Control and corticosterone; **Time:** pre-experiment and post-experiment; **Type III SS:** Type III sum of squares; **DF:** Degrees of freedom; **MS:** Mean square. Variables with *P* significant < 0.05 are highlighted in bold. Experiment details: **Exp. 2:** 0h *vs*. 1h *vs*. 24h restraint; **Exp. 5:** field *vs*. 7 *vs*. 30 *vs*. 60 *vs*. 90 days in captivity; **Exp. 6:** corticosterone transdermal application.
