# Supplementary material for: Time-related immunomodulation by stressors and corticosterone transdermal application in toads
Source: PLoS One. 2019 Sep 20;14(9):e0222856. doi: 10.1371/journal.pone.0222856 (PMC6754171; doi:10.1371/journal.pone.0222856)
Supplement: S9 Table — Effect of Restraint Challenge (Exp. 2) and captivity duration (Exp. 5) on phagocytosis of R. ornata tested through a set of univariate ANOVAs, with phagocytosis percentage and phagocytosis efficiency as dependent variables and time (0, 1 and 24h) and captivity duration (field, 7, 30, and 90 days) as factors. (DOCX) [file pone.0222856.s009.docx]

**Table S9. Phagocytosis percentage analysis of variance after corticosterone transdermal application in *R. ornata* toads**. Effect of Restraint Challenge (Exp. 2) and captivity duration (Exp. 5) on phagocytosis of *R. ornata* tested through a set of univariate ANOVAs, with phagocytosis percentage and phagocytosis efficiency as dependent variables and hour (0, 1 and 24h) and captivity duration (field, 7, 30, and 90 days) as factors.

| **Experiment** | **Variable** | **Source** | **Type III SS** | **DF** | **MS** | **F** | ***P*** |
| --- | --- | --- | --- | --- | --- | --- | --- |
| **Experiment 2:**  **Restraint**  **0 *vs.* 1 *vs.* 24h**  **(ANOVA)** | **Phagocytosis Percentage (%)** | Intercept | 140.209 | 1 | 140.209 | 24.456 | **0.008** |
|  |  | Error | 22.932 | 4 | 5,733 |  |  |
|  |  | Hour | 0.548 | 1 | 0.383 | 0.055 | 0.897 |
|  |  | Error (Hour) | 39.909 | 6 | 6.978 |  |  |
| **Experiment 5:**  **Captivity Duration**  **(univariate ANOVA)** | **Phagocytosis Percentage (%)** | Intercept | 1.225.081 | 1 | 1.225.081 | 50.595 | **≤ 0.001** |
|  |  | CD (days) | 171.814 | 2 | 85.907 | 3.548 | **0.053** |
|  |  | Error | 387.415 | 16 | 24.213 |  |  |
|  |  | Total | 1.858.032 | 19 |  |  |  |
|  |  | Corrected Total | 559.229 | 18 |  |  |  |

Abbreviation as follow: **Hour:** 0, 1, 24h; **CD:** Captivity duration; **Type III SS:** Type III sum of squares; **DF:** Degrees of freedom; **MS:** Mean square. Variables with *P* significant < 0.05 are highlighted in bold. Experiment details: **Exp. 2:** 0h *vs*. 1h *vs*. 24h restraint; **Exp. 5:** field *vs*. 7 *vs*. 30 *vs*. 90 days in captivity.
