# Supplementary material for: Time-related immunomodulation by stressors and corticosterone transdermal application in toads
Source: PLoS One. 2019 Sep 20;14(9):e0222856. doi: 10.1371/journal.pone.0222856 (PMC6754171; doi:10.1371/journal.pone.0222856)
Supplement: S10 Table — Effect of corticosterone transdermal application (Exp. 6) on phytohemagglutinin relative edema of R. ornata tested through a set of mixed ANCOVAs, with phytohemagglutinin relative edema as dependent variable, body mass as co-variable and time (12 and 24 hours post injection) and group (control and corticosterone) as factors. (DOCX) [file pone.0222856.s010.docx]

**Table S10.** **Phytohemagglutinin relative edema analysis of variance after corticosterone transdermal application in *R. ornata* toads.** Effect of corticosterone transdermal application (Exp. 6) on phytohemagglutinin relative edema of *R. ornata* tested through a set of mixed ANCOVAs, with phytohemagglutinin relative edema as dependent variable, body mass as co-variable and time (12 and 24 hours post injection) and group (control and corticosterone) as factors.

| **Variable** | **Source** | **Type III SS** | **DF** | **MS** | **F** | ***P*** |
| --- | --- | --- | --- | --- | --- | --- |
| **Relative Edema (%)** | Intercept | 181.302 | 1 | 181.302 | 3.884 | 0.066 |
|  | Body Mass | 245.205 | 1 | 245.205 | 5.253 | **0.036** |
|  | Group | 400.835 | 1 | 400.835 | 8.587 | **0.010** |
|  | Error (Group) | 746.908 | 16 | 46.682 |  |  |
|  | Time | 5.582 | 1 | 5.582 | 0.168 | 0.687 |
|  | Time * Body Mass | 3.233 | 1 | 3.233 | 0.097 | 0.759 |
|  | Time * Group | 2.129 | 1 | 2.129 | 0.064 | 0.803 |
|  | Error (Time) | 531.133 | 16 | 33.196 |  |  |

Abbreviation as follow: **Group:** Control and corticosterone; **Time:** pre-experiment and post-experiment; **Type III SS:** Type III sum of squares; **DF:** Degrees of freedom; **MS:** Mean square. Variables with *P* significant < 0.05 are highlighted in bold. Experiment details: **Exp. 6:** corticosterone transdermal application.
