# Supplementary material for: Time-related immunomodulation by stressors and corticosterone transdermal application in toads
Source: PLoS One. 2019 Sep 20;14(9):e0222856. doi: 10.1371/journal.pone.0222856 (PMC6754171; doi:10.1371/journal.pone.0222856)
Supplement: S1 File — File containing full raw data of the six performed experiments in this study. (DOCX) [file pone.0222856.s011.docx]

Data Availability File for

**Time-related immunomodulation by stressors and corticosterone transdermal application in toads**

Stefanny Christie Monteiro Titon^*^, Braz Titon Junior, Adriana Maria Giorgi Barsotti, Fernando Ribeiro Gomes, Vania Regina Assis

Departamento de Fisiologia, Instituto de Biociências, Universidade de São Paulo, São Paulo, SP, Brasil

^*^Corresponding author: [stefannychristie@gmail.com](mailto:stefannychristie@gmail.com)

**File containing full raw data of the six performed experiments in this study.**

**Raw data for the experiment 1 (Restraint challenge: field *vs*. 24h).**

| **Individual** | **Body Mass**  **(g)** | **SVL**  **(mm)** | **Hour (Restraint)** | **CORT**  **(ng/ml)** | **NLR** | **BKA**  **(%)** |
| --- | --- | --- | --- | --- | --- | --- |
| 1 | 18.82 | 62.82 | Field | 71.83 | 0.93 | 54 |
|  |  |  | 24h Restraint | 66.54 | 3.00 | 100 |
| 2 | 16.17 | 60.25 | Field | 142.64 | 0.70 | 100 |
|  |  |  | 24h Restraint | 33.11 | 1.26 | 73 |
| 3 | 17.09 | 59.10 | Field | 28.28 | 0.56 | 98 |
|  |  |  | 24h Restraint | 47.25 | 1.00 | 100 |
| 4 | 19.63 | 62.50 | Field | 114.21 | 0.32 | 100 |
|  |  |  | 24h Restraint | 56.81 | 1.69 | 100 |
| 5 | 14.14 | 60.67 | Field | 46.02 | 0.24 | 100 |
|  |  |  | 24h Restraint | 60.66 | 2.48 | 78 |
| 6 | 18.69 | 63.83 | Field | 108.80 | 0.47 | 100 |
|  |  |  | 24h Restraint | 47.93 | 2.81 | 98 |
| 7 | 15.61 | 59.25 | Field | 105.15 | 0.09 | 96 |
|  |  |  | 24h Restraint | 18.79 | 0.45 | 90 |
| 8 | 18.83 | 65.15 | Field | 8.14 | 0.91 | 44 |
|  |  |  | 24h Restraint | 1.47 | 0.15 | 0 |
| 9 | 14.54 | 59.88 | Field | 20.27 |  | 4 |
|  |  |  | 24h Restraint | 2.95 |  | 0 |
| 10 | 15.58 | 55.96 | Field | 5.78 | 0.31 | 100 |
|  |  |  | 24h Restraint | 27.44 | 5.14 | 96 |

Abbreviation as follow: **SVL:** Snout-Vent Length; **CORT:** plasma corticosterone levels; **NLR:** neutrophil:lymphocyte ratio; **BKA:** bacterial killing ability.

**Raw data for the experiment 2 (Restraint challenge: 0h *vs*. 1h *vs*. 24h).**

| **Individual** | **Body Mass**  **(g)** | **SVL**  **(mm)** | **Hour (Restraint)** | **CORT**  **(ng/ml)** | **BKA**  **(%)** | **PP**  **(%)** |
| --- | --- | --- | --- | --- | --- | --- |
| 1 | 13.92 | 61.13 | 0h Restraint | 1.22 | 100 | 1.25 |
|  |  |  | 1h Restraint | 182.84 | 84 | 2.62 |
|  |  |  | 24h Restraint | 9.07 | 100 | 1.50 |
| 2 | 15.35 | 58.68 | 0h Restraint | 7.03 | 83 | 4.92 |
|  |  |  | 1h Restraint | 219.03 | 78 | 1.88 |
|  |  |  | 24h Restraint | 38.44 | 95 | 4.11 |
| 3 | 12.19 | 55.12 | 0h Restraint | 2.41 | 0 | 1.18 |
|  |  |  | 1h Restraint | 62.37 | 0 | 6.25 |
|  |  |  | 24h Restraint | 17.95 | 0 | 7.41 |
| 4 | 15.92 | 58.17 | 0h Restraint | 4.29 | 0 | 1.36 |
|  |  |  | 1h Restraint | 207.98 | 63 | 1.38 |
|  |  |  | 24h Restraint | 40.33 | 0 | 2.11 |
| 5 | 11.65 | 52.81 | 0h Restraint | 7.54 | 83 |  |
|  |  |  | 1h Restraint | 40.00 | 24 | 4.23 |
|  |  |  | 24h Restraint | 69.04 | 49 | 1.05 |
| 6 | 15.33 | 57.51 | 0h Restraint | 0.32 | 38 | 6.10 |
|  |  |  | 1h Restraint | 1.79 | 92 | 2.30 |
|  |  |  | 24h Restraint | 52.15 | 55 | 1.49 |
| 7 | 13.29 | 57.00 | 0h Restraint | 9.73 | 98 | 6.70 |
|  |  |  | 1h Restraint | 14.35 | 96 |  |
|  |  |  | 24h Restraint | 9.81 | 96 | 9.09 |

Abbreviation as follow: **SVL:** Snout-Vent Length; **CORT:** plasma corticosterone levels; **BKA:** bacterial killing ability; **PP:** phagocytosis percentage.

**Raw data for the experiment 3 (Short -term Captivity: Field *vs*. 7d).**

| **Individual** | **Body Mass**  **(g)** | **SVL**  **(mm)** | **Captivity (days)** | **CORT**  **(ng/ml)** | **NLR** | **BKA**  **(%)** |
| --- | --- | --- | --- | --- | --- | --- |
| 1 | 18.88 | 64.98 | Field | 6.16 | 0.12 | 0 |
|  |  |  | 7 days captivity | 3.42 | 0.19 | 0 |
| 2 | 14.72 | 58.84 | Field | 7.86 | 0.42 | 23 |
|  |  |  | 7 days captivity | 21.09 | 0.09 | 87 |
| 3 | 15.23 | 60.13 | Field | 8.05 | 0.66 | 92 |
|  |  |  | 7 days captivity | 1.83 | 0.07 | 83 |
| 4 | 11.24 |  | Field | 18.26 | 0.13 | 0 |
|  |  |  | 7 days captivity | 10.45 | 0.02 | 0 |
| 5 | 12.85 | 56.42 | Field | 5.11 | 0.05 | 0 |
|  |  |  | 7 days captivity | 10.70 | 0.26 | 90 |
| 6 | 19.15 | 64.09 | Field | 8.57 |  | 92 |
|  |  |  | 7 days captivity | 14.16 |  | 0 |
| 7 | 20.64 | 64.60 | Field | 5.01 | 0.40 | 96 |
|  |  |  | 7 days captivity | 1.55 | 0.40 | 0 |

Abbreviation as follow: **SVL:** Snout-Vent Length; **CORT:** plasma corticosterone levels; **NLR:** neutrophil:lymphocyte ratio; **BKA:** bacterial killing ability.

**Raw data for the experiment 4 (Short-term Captivity: Field *vs*. 30d).**

| **Individual** | **Body Mass**  **(g)** | **Captivity (days)** | **CORT**  **(ng/ml)** | **NLR** | **BKA**  **(%)** |
| --- | --- | --- | --- | --- | --- |
| 1 | 11.47 | Field | 27.87 |  | 0 |
|  |  | 30 days captivity |  |  |  |
| 2 | 14.56 | Field | 41.21 |  | 7 |
|  |  | 30 days captivity |  |  |  |
| 3 | 13.85 | Field | 31.26 | 0.57 | 43 |
|  |  | 30 days captivity | 21.40 | 0.70 | 0 |
| 4 | 16.97 | Field | 26.53 |  | 81 |
|  |  | 30 days captivity | 37.11 |  | 84 |
| 5 | 15.93 | Field | 41.72 | 0.31 | 60 |
|  |  | 30 days captivity | 17.59 | 0.30 | 13 |
| 6 | 12.74 | Field | 37.99 | 0.27 | 0 |
|  |  | 30 days captivity | 11.81 | 0.19 | 11 |
| 7 | 13.54 | Field | 10.18 | 0.16 | 51 |
|  |  | 30 days captivity | 13.26 | 0.20 | 0 |
| 8 | 14.12 | Field | 7.31 | 0.29 | 7 |
|  |  | 30 days captivity | 16.92 | 0.17 | 63 |
| 9 | 17.44 | Field | 36.60 | 1.72 | 85 |
|  |  | 30 days captivity | 9.47 | 0.12 | 34 |
| 10 | 11.23 | Field | 53.87 | 0.08 | 31 |
|  |  | 30 days captivity | 21.09 | 0.14 | 0 |

Abbreviation as follow: **SVL:** Snout-Vent Length; **CORT:** plasma corticosterone levels; **NLR:** neutrophil:lymphocyte ratio; **BKA:** bacterial killing ability.

**Raw data for the experiment 5 (Long-term Captivity: Field *vs*. 7d *vs*. 30d *vs*. 60d *vs*. 90d).**

| **Individual** | **Body Mass**  **(g)** | **SVL**  **(mm)** | **Captivity duration (days)** | **CORT**  **(ng/ml)** | **BKA**  **(%)** | **PP**  **(%)** |
| --- | --- | --- | --- | --- | --- | --- |
| 1 | 12.85 | 57.41 | Field | 62.43 | 92 |  |
| 2 | 17.59 | 64.36 |  | 33.46 | 94 |  |
| 3 | 16.17 | 60.82 |  | 46.51 | 93 |  |
| 4 | 15.61 | 61.44 |  | 38.96 | 94 |  |
| 5 | 18.42 | 62.10 |  | 22.46 | 94 |  |
| 6 | 10.83 | 52.72 |  | 45.09 |  |  |
| 7 | 19.11 | 60.45 |  | 38.92 |  |  |
| 8 | 16.32 | 59.28 |  | 30.71 |  |  |
| 9 | 17.45 | 63.88 |  | 55.08 | 97 |  |
| 10 | 13.86 | 57.56 |  | 23.08 | 91 |  |
| 11 | 16.65 | 61.58 |  | 10.92 | 93 |  |
| 12 | 14.75 | 57.41 | 7 days captivity | 10.45 | 0 | 7.39 |
| 13 | 18.84 | 64.36 |  | 9.17 | 80 | 9.64 |
| 14 | 12.07 | 57.30 |  |  |  | 7.66 |
| 15 | 15.49 | 61.44 |  | 12.13 | 86 | 3.86 |
| 16 | 16.10 | 63.15 |  | 10.59 | 0 | 6.89 |
| 17 | 11.90 | 57.41 |  | 2.03 |  | 3.47 |
| 18 | 16.53 | 65.39 |  | 7.63 | 100 |  |
| 19 | 14.88 | 60.82 | 30 days captivity | 17.92 | 74 | 15.34 |
| 20 | 14.58 | 61.44 |  | 14.02 | 67 | 3.16 |
| 21 | 11.35 | 58.95 |  | 12.41 | 85 | 21.52 |
| 22 | 15.00 | 61.58 |  | 13.33 | 87 | 7.61 |
| 23 | 12.49 | 60.37 |  | 10.99 | 80 | 12.25 |
| 24 | 14.80 | 63.82 |  | 2.13 | 78 | 20.73 |
| 25 | 10.46 | 58.50 |  | 10.15 | 87 | 4.59 |
| 26 | 14.86 | 62.10 | 60 days captivity | 13.19 | 86 |  |
| 27 | 7.55 | 52.72 |  | 12.47 | 72 |  |
| 28 | 9.05 | 55.22 |  |  |  |  |
| 29 | 13.37 | 61.48 |  | 6.42 |  |  |
| 30 | 13.60 | 58.90 |  | 2.18 | 85 |  |
| 31 | 15.30 | 61.46 |  | 9.33 |  |  |
| 32 | 15.55 | 59.18 |  | 9.51 | 92 |  |
| 33 | 16.26 | 60.45 | 90 days captivity |  |  | 4.57 |
| 34 | 11.58 | 59.28 |  | 5.76 | 0 | 7.23 |
| 35 | 14.29 | 60.59 |  | 6.97 | 89 |  |
| 36 | 16.52 | 63.88 |  |  |  | 2.97 |
| 37 | 11.11 | 57.56 |  | 4.86 | 33 | 7.95 |
| 38 | 11.82 | 58.36 |  | 5.01 | 7 |  |
| 39 | 11.15 | 57.20 |  |  |  | 7.62 |
| 40 | 11.21 | 57.25 |  |  |  | 2.64 |

Abbreviation as follow: **SVL:** Snout-Vent Length; **CORT:** plasma corticosterone levels; **BKA:** bacterial killing ability; **PP:** phagocytosis percentage.

**Raw data for the experiment 6 (Corticosterone Transdermal application).**

| **Ind** | **Body Mass**  **(g)** | **SVL**  **(mm)** | **Group** | **Pre-Treatment** | | | **Post-Treatment** | | | | |
| --- | --- | --- | --- | --- | --- | --- | --- | --- | --- | --- | --- |
|  |  |  |  | **CORT**  **(ng/ml)** | **NLR** | **BKA**  **(%)** | **CORT**  **(ng/ml)** | **NLR** | **BKA**  **(%)** | **Edema 12h** | **Edema 24h** |
| 1 | 64.98 | 18.88 | Control | 3.42 | 0.19 | 0 | 32.99 | 0.09 | 0 | 5.46 | 2.53 |
| 2 |  | 11.24 |  | 10.45 | 0.02 | 0 |  |  |  |  |  |
| 3 | 64.60 | 20.64 |  | 1.55 | 0.40 | 0 | 17.63 | 0.21 | 93 | 7.68 | 1.54 |
| 4 | 53.17 | 12.12 |  | 1.37 | 0.28 | 69 | 36.44 | 0.57 | 90 | 0.89 | 8.53 |
| 5 | 61.93 | 18.20 |  | 17.77 | 0.63 | 75 | 22.52 | 0.88 | 38 | 18.29 | 2.39 |
| 6 | 55.44 | 13.06 |  | 8.77 | 0.19 | 0 | 21.05 | 0.21 | 0 | 2.67 | -3.91 |
| 7 | 61.56 | 15.52 |  | 17.18 | 0.08 | 0 | 4.04 | 0.51 | 0 | 11.48 | 14.64 |
| 8 | 60.13 | 15.23 | CORT | 1.83 | 0.07 | 83 | 396.53 | 0.10 | 27 | 9.46 | -7.20 |
| 9 | 64.09 | 19.15 |  | 14.16 |  | 0 | 372.55 | 0.11 | 91 | 2.15 | 1.64 |
| 10 | 60.63 | 14.72 |  | 3.91 | 0.16 | 0 |  | 0.50 |  | -14.62 | -25.73 |
| 11 | 66.69 | 20.66 |  | 32.34 | 0.18 | 56 | 602.01 | 0.15 | 0 | -0.42 | -3.57 |
| 12 | 63.77 | 21.31 |  | 26.10 | 0.14 | 10 | 175.05 | 0.44 | 11 | 5.82 | -8.27 |
| 13 | 62.28 | 16.91 |  | 24.95 | 0.24 | 0 | 255.92 | 0.44 | 0 | 14.53 | 19.27 |
| 14 | 51.48 | 11.06 |  | 15.88 | 0.19 | 0 | 276.51 | 0.41 | 0 | -3.27 | -12.25 |
| 15 |  | 12.55 |  | 20.20 | 0.07 | 0 |  |  |  |  |  |
| 16 | 59.54 | 14.37 |  | 35.31 | 0.19 | 98 | 525.15 | 0.15 | 84 | 0.82 | -10.48 |
| 17 | 69.71 | 20.79 |  | 5.14 | 0.07 | 58 | 184.31 | 0.11 | 0 | 9.26 | 2.62 |
| 18 | 58.84 | 14.72 | Placebo | 21.09 | 0.09 | 87 | 31.35 | 0.18 | 0 | 12.56 | 12.95 |
| 19 | 56.42 | 12.85 |  | 10.70 | 0.26 | 90 | 27.29 | 0.05 | 91 | -2.11 | 0.29 |
| 20 | 62.68 | 15.09 |  |  | 0.34 |  | 33.39 | 0.26 |  | 11.88 | -1.37 |
| 21 | 55.80 | 15.07 |  | 5.81 | 0.43 |  | 90.49 | 0.63 | 64 | 0.36 | -0.64 |
| 22 | 57.87 | 14.75 |  |  |  |  |  | 0.45 |  | -2.15 | -11.25 |
| 23 | 54.59 | 12.75 |  | 17.46 | 0.09 | 0 | 9.71 | 0.31 | 0 | 11.37 | -1.59 |
| 24 | 58.24 | 14.41 |  | 8.60 | 0.17 | 0 | 42.17 | 1.15 | 0 | 16.79 | -6.97 |

Abbreviation as follow: **SVL:** Snout-Vent Length; **CORT:** plasma corticosterone levels; **NLR:** neutrophil:lymphocyte ratio, **BKA:** bacterial killing ability.
