# Supplementary material for: Time-related immunomodulation by stressors and corticosterone transdermal application in toads
Source: PLoS One. 2019 Sep 20;14(9):e0222856. doi: 10.1371/journal.pone.0222856 (PMC6754171; doi:10.1371/journal.pone.0222856)
Supplement: Attachment — Submitted filename: Response to reviewers PONE-D-19-16526.docx [file pone.0222856.s012.docx]

**Response to reviewers**

(Please note the lines indicated in this letter are related to the manuscript with track changes)

**Reviewer #1**

307: Please explain why the sampling dates (years) are spreaded so much? 308: Why sampling was done across different locations?

- This paper results from studies associated with 3 different Ph.D projects (Stefanny C. M. Titon, Adriana M. G. Barsotti and Vania R. Assis). In each project, different questions concerning hormonal and immune changes following stressors were addressed in the species *Rhinella ornata*, and some common points were raised. When we observed the results from these different projects, interesting patterns in terms of acute and chronic immunomodulation emerged. We then decided to write only one paper, bringing together all these results. For this reason, we have different years and collections’ site. The procedures used to collect all the data sets (Restraint challenge: experiment 1 and 2; Captivity: experiment 3, 4 and 5) are standard methodologies in our Lab, so we can assure they were all ran under the same conditions throughout the years. In the meantime, although the collections’ sites were different, the climatic variations were similar between them and the animals demonstrates the reproductive behavior at the same period of the year in the three locations. Therefore, the study of general patterns in physiological variables, within a single species, from different locations along 6 years, brings important information about how these toads respond to stressors.

313: I can see ethics approval for 2013 and 2016 only. Did this ethics approval cover the 2018 sampling as well?

- Brazilian Ethics committee approves projects that encompasses many years. They have a protocol number that indicates the year of approval and the process number of the year. We indicate in the manuscript only the numbers of the processes: CEUA n° 142/2011; 054/2013; 249/2016 (Lines: 315-316). However, these projects encompassed the following years: CEUA n° 142/2011 (2011-2015); 054/2013 (2013-2017) and 249/2016 (2016-2019). In our manuscript we reported six different experiments, which were all approved by the Ethic committee of the Biosciences Institute of University of Sao Paulo as follows:
- CEUA n° 142/2011 - Experiment 1 Restraint (field vs. 24h), Experiment 3 Captivity (Short-term captivity field vs. 7 days), Experiment 6 Corticosterone transdermal application (pre vs. post treatment - control, placebo, corticosterone);
- CEUA n° 054/2013 - Experiment 5 Captivity (Long-term captivity field vs. 7 vs. 30 vs. 60 vs. 90 days); and
- CEUA n° 249/2016 - Experiment 2 Restraint (0h vs. 1h. vs. 24h), Experiment 4 Captivity (Mid-term captivity (field vs. 30 days).

**Reviewer #2**

Methods

Line 316. Correct “toads were located by visually and captured by hand”.

- We adjusted the sentence as follows: Toads were visually located and hand captured. (Line 319).

The relationship between body mass and CORT was examined but was body size measured? I’m wondering how the authors accounted for differences in mass that were attributable to size. For example, there was no relationship between body mass and CORT (Line 359 and elsewhere) but would it be better to examine body condition estimates that factor in size differences?

- We thank the reviewer for bringing this point. Yes, we measured all the animals with a digital caliper (0.01mm). We didn't have this information in the manuscript originally submitted, but we added this information in this version.
- Since the reviewer bring this point, we included the snout-vent length and also calculated a body index as the residual of the regression of the body mass as a function of the body size and included in the analyses.
- We included both variables in the manuscript as follows:

*Methods section*:

Lines 321-322: “All the animals were weighed…. and measured (to the nearest 0.01mm) after capture.”

*Statistical analysis section*:

Lines: 468-470: “…body mass and snout-vent length as co-variables and each treatment and/or time as a factor. Unstandardized residuals of a linear regression of body mass as a function of snout-vent length were used as body index and also included as co-variable in the ANCOVAs.”

Lines: 471-472: “…For the dependent variables not significantly affected by body mass, snout-vent length and body index a set of ANOVAs…”

*Results section*:

Lines: 99-103: “Except for PHA relative edema, which was affected by body mass and snout-vent length (*P* = 0.036 and *P* < 0.001, respectively), morphological measures (body mass, snout-vent length and body index) did not affect any other studied variable at any circumstance: NLR (*P* ≥ 0.193); BKA (*P* ≥ 0.101); PP (*P* ≥ 0.062); CORT plasma levels (*P* ≥ 0.193); PHA (*P* ≥ 0.541, for body index).”

*We also included the following statement* (Lines: 182-183): “There were no differences in body mass and snout-vent length between treatment groups (t_17_ = -1.245, *P* = 0.230 and t_17_ = -1.540, *P* = 0.142, respectively).”

Line 451. How many CORT assays were run? Were the assays validated for this species?

- In order to measure plasma CORT levels of all individuals from the 6 experiments we ran 10 CORT assays. The inter-assay variation was 4.77% and includes all the assays, as cited in the manuscript (Line 458).
- The CORT assay was not validated for this particular species. However, this assay is a standard technique in our lab, and we validated this assay for 3 different anuran species, including another toad species, *Rhinella icterica* (Assis et al., 2017), and two different treefrogs, *Boana albopunctata* (Titon et al., 2016) and *Boana faber* (Barsotti et al., 2017). We have been using this CORT kit in our Lab to investigate plasma corticosterone levels in anurans under field conditions, as well as, in those individuals subjected to restraint challenge and corticosterone transdermal application. Also, considering the hormone data we are showing in this manuscript, we can observe how wide is the corticosterone hormone levels range that we can measure with Cayman kit. The kit is reliable to measure levels as low as 0.32ng/ml (dilution 1:4), found in the experiment 2 (individual of 0h), as well as high like 602,01 (dilution 1:600), found on experiment 6 (individual from experimental group of the CORT transdermal application experiment). We also would like to mention that the kit is sensitive to detect increases in corticosterone levels after the animals were submitted to a challenge or hormone application, when comparing the animals with themselves (before and after) or different groups (placebo and CORT-treated). Finally, our research group have published several other papers using this same kit: (Assis et al., 2019, 2017, 2015; Barsotti et al., 2017; Madelaire et al., 2019, 2017; Titon et al., 2018).

Results

Line 99. Body mass was not manipulated so it would be better to state that there was no relationship between body mass and the measured variables. What was the direction of the relationship with PHA?

- We appreciate the reviewer suggestion and adjusted the sentence: “… there were no relationship between morphological measures (body mass, snout-vent length and body index) and any other studied variable at any circumstance…” (Lines: 100-102).
- There was a direct relationship between both, body mass and snout-vend length, and PHA edema independently of time (12 or 24h). We included this information in the manuscript: “Except for PHA relative edema, which was positively affected by body mass and snout-vent length…” (Lines: 99-100).

Line 102. How were toads with high and low initial CORT levels delineated (i.e., how did you determine the cutoff point)?

- The cutoff point was based on the CORT levels found associated with calling behavior. During calling behavior amphibian anurans exhibit high CORT levels (Leary et al., 2004; Titon et al., 2018). Therefore, the CORT levels equal or higher to those found in calling toads at the moment of capture (≥ 44.30 ± 33.24; mean ± standard deviation) were defined as high, and values equal or lower to those found on non-calling toads defined as low (≤ 6.54 ± 4.39; mean ± standard deviation). We included a sentence in the result section: Toads displaying high CORT levels in the field (those exhibiting calling behavior at the moment of capture [27])…” (Lines: 104-105).
- This topic is also pointed in the discussion section: “Meanwhile, for the individuals in the field displaying high baseline CORT... These toads were already displaying high CORT in the field probably due to reproductive behavior (calling activity) [27], precluding the assessment of increased CORT values 24h post-restraint [11].” (Lines: 209-211).

Line 111. Rephrase to state that CORT levels were 14 times higher in TA-treated toads compared to controls.

- We rephased the sentence as suggested. (Lines: 113-115).

CORT levels increased dramatically in TA-treated toads (Fig 1E). The authors mention the upper limits of CORT in this species following severe dehydration (line 221), but can the authors comment about the natural range of CORT levels and whether the effects on immune responses (especially PHA measures shown in Fig. 4C) actually occur within the natural range of CORT levels (i.e., CORT levels in the TA treatment group is still quite a bit higher than the upper limits reported)?

- Regarding natural variations in CORT plasma levels, the higher CORT values we have reported for *R. ornata* species are those associated with calling behavior (higher value: 62.43 ng/ml, in this present study; and 142.64ng/ml (Assis et al., 2019).
- In the present study, we used CORT transdermal application in order to promote daily acute increases in CORT plasma levels, which resulted in such high CORT levels (mean values 350 ng/ml), and generated immunosuppressive effects. It would be certainly interesting to repeat this transdermal application using lower doses, in order to achieve CORT plasma levels more commonly observed for this species in ecologically relevant contexts. Our lab students are also still performing studies on natural variation in CORT plasma levels and trying to mimic those levels through CORT transdermal application for this and other species of *Rhinella*.

Line 125. Of the 9 individuals wherein NLR was measured after constraint (Fig. 2A), do these represent individuals that showed an increase or decrease in CORT levels during constraint (see Fig. 1 A and B)?

- The individuals of the figure 2A, are the same of the figure 1A as indicated by the legend in the figures: Exp. 1: Restraint. These individuals showed increased NLR, but decreased CORT levels in response to restraint.

**Data availability statement:**

Data availability

- We included full data in the supplementary file as tables S11-S16. (Indicated in Lines: 673-679).

**References cited in this letter**

Assis, V.R., Monteiro Titon, S.C., Teixeira Queiroz-Hazarbassanov, N.G., de Oliveira Massoco, C., Ribeiro Gomes, F., 2017. Corticosterone transdermal application in toads (Rhinella icterica): Effects on cellular and humoral immunity and steroid plasma levels. J. Exp. Zool. Part A Ecol. Integr. Physiol. 327, 200–213. https://doi.org/10.1002/jez.2093

Assis, V.R., Titon, S.C.M., Barsotti, A.M.G., Titon, B., Gomes, F.R., 2015. Effects of acute restraint stress, prolonged captivity stress and transdermal corticosterone application on immunocompetence and plasma levels of corticosterone on the cururu toad (Rhinella icterica). PLoS One 10, 1–21. https://doi.org/10.1371/journal.pone.0121005

Assis, V.R., Titon, S.C.M., Gomes, F.R., 2019. Acute stress, steroid plasma levels, and innate immunity in Brazilian toads. Gen. Comp. Endocrinol. 273, 86–97. https://doi.org/10.1016/j.ygcen.2018.05.008

Barsotti, A.M.G., de Assis, V.R., Titon, S.C.M., Titon, B., da Silva Ferreira, Z.F., Gomes, F.R., 2017. ACTH modulation on corticosterone, melatonin, testosterone and innate immune response in the tree frog Hypsiboas faber. Comp. Biochem. Physiol. -Part A Mol. Integr. Physiol. 204, 177–184. https://doi.org/10.1016/j.cbpa.2016.12.002

Leary, C.J., Jessop, T.S., Garcia, A.M., Knapp, R., 2004. Steroid hormone profiles and relative body condition of calling and satellite toads: Implications for proximate regulation of behavior in anurans. Behav. Ecol. 15, 313–320. https://doi.org/10.1093/beheco/arh015

Madelaire, C.B., Cassettari, B. de O., Gomes, F.R., 2019. Immunomodulation by testosterone and corticosterone in toads: Experimental evidences from transdermal application. Gen. Comp. Endocrinol. 273, 227–235. https://doi.org/10.1016/j.ygcen.2018.09.005

Madelaire, C.B., Sokolova, I., Gomes, F.R., 2017. Seasonal Patterns of Variation in Steroid Plasma Levels and Immune Parameters in Anurans from Brazilian Semiarid Area. Physiol. Biochem. Zool. 90, 415–433. https://doi.org/10.1086/691202

Titon, S.C.M., Assis, V.R., Titon Jr, B., Barsotti, A.M.G., Flanagan, S.P., Gomes, F.R., 2016. Calling rate, corticosterone plasma levels and immunocompetence of Hypsiboas albopunctatus. Comp. Biochem. Physiol. -Part A Mol. Integr. Physiol. 201, 53–60. https://doi.org/10.1016/j.cbpa.2016.06.023

Titon, S.C.M., Titon Junior, B., Assis, V.R., Kinker, G.S., Fernandes, P.A.C.M., Gomes, F.R., 2018. Interplay among steroids, body condition and immunity in response to long-term captivity in toads. Sci. Rep. 8, 1–13. https://doi.org/10.1038/s41598-018-35495-0
